# Mental Stigma Among Al-Ahsa Population in Saudi Arabia

**DOI:** 10.7759/cureus.19710

**Published:** 2021-11-18

**Authors:** Mohammed Alamer, Ali Alsaad, Mohammed Al-Ghareeb, Almukhtar Almomatten, Mohammed Alaethan, Mohammed A AlAmeer

**Affiliations:** 1 Psychiatry, College of Medicine, King Faisal University, Al-Ahsa, SAU; 2 Clinical Neuroscience, College of Medicine, King Faisal University, Al-Ahsa, SAU

**Keywords:** public psychiatry, psychiatric patient, stigma, mental illness, mental stigma

## Abstract

Introduction

Mental stigma is when patients of mental illness are labeled by their disorders, viewed negatively, and discriminated against due to their illness. This has a big impact on people's attitudes and behaviors toward the mentally ill. Moreover, mental stigma leads people to avoid patients with mental health issues, blame them for their conditions, and associate them with negative features like being dangerous, unpredictable, and hard to communicate with. The primary objective of this study is to assess the mental stigma among the population of the Al-Ahsa region in Saudi Arabia.

Methods

This is a cross-sectional study conducted in Al-Ahsa, Saudi Arabia, between June and August 2021. The target population of the study was people aged 18-65 years old whose place of residency during the time of the study was Al-Ahsa. The questionnaire used in this study contained a mental stigma scale called community attitudes toward mental illness (CAMI).

Results

A total of 758 participants were enrolled in the study. The mean of the stigma score was 99.24 ± 15.622, with a minimum of 53 and a maximum of 176. The means of the subscales were as follows: authoritarianism (26.18 ± 4.420), benevolence (23.26 ± 4.762), community mental health ideology (24.66 ± 5.896), and social restrictiveness (25.13 ± 4.6). There was a positive relationship between age and stigma score. Healthcare workers were found to have a lower level of stigma, as were those who spoke more languages. Also, being diagnosed with a mental illness and knowing someone who is diagnosed with a mental illness were associated with a lower level of stigma.

Conclusion

Most of the population (91.96%) had a low to medium-low level of stigma. The variables found to affect the level of stigma were age, career, number of spoken languages, being diagnosed with a mental illness, and knowing someone who is diagnosed with a mental illness. Mental stigma has a great impact on the person, as it can prevent the patient from seeking help, lead to isolation, and suicidal ideation. Future stigma-targeted campaigns are recommended.

## Introduction

Psychiatric disorders are mental disorders of the brain that can cause disturbance of thoughts, behaviors, and emotions [1]. The prevalence of common mental disorders is high and can affect people worldwide [2,3]. Despite the high global prevalence of psychiatric disorders, the literature has shown that the awareness and attitude of the population towards these disorders is not adequate in developing countries, while it is still improving in developed counties [2-9]. It has been shown in different studies that people with mental illness are highly stigmatized [10,11]. Mental stigma is when patients of mental illness are labeled by their disorders, viewed negatively, and discriminated against due to their illness. This has a big impact on people's attitudes and behaviors towards the mentally ill [11-13]. Moreover, mental stigma leads people to avoid mental illness patients, blame them for their conditions, and associate them with negative features like being dangerous, unpredictable, and hard to communicate with [10,11].

A major issue with mental stigma is its effect on multiple aspects of the patient’s life and on the prognosis of the disease itself. It prevents patients from seeking help and delays treatment and recovery. Also, it leads to isolation and unemployment of the patient [11,14,15]. The effect of stigma does not come from society alone but also from healthcare workers. Patients with mental illness may get less care due to the presumptuous association of their mental illness and physical symptoms, and the negative attitude healthcare workers might show towards these patients, which leads to the feeling of being unwelcome in healthcare facilities [13].

In Saudi Arabia, the number of new and recurrent cases in outpatient clinics at psychiatric hospitals and psychiatric departments of other hospitals in 2017 was 397,210 cases (which is about 1.22% of the entire population), 43,846 of which were admitted, while the number of cases in 2016 was 462,282 patients (1.46%). In 2012, it was 495,484 (1.7%) [16-18]. Despite the burden of mental illness in Saudi Arabia, studies have shown that the Saudi population has insufficient knowledge and attitude toward mental illness [14,15,19-22].

The primary objective of this study is to assess the mental stigma among the population of Al-Ahsa region in Saudi Arabia. The hypothesis is that there is a strong stigma among the targeted population.

## Materials and methods

Study setting and population

This is a cross-sectional study conducted in the Al-Ahsa region of Saudi Arabia between June and August 2021. The target sample size of this study was 812 participants, based on a recent study that was conducted in the Al-Ahsa region targeting the same population [23]. The goal of the study was explained to the participants. The data were completely anonymous and confidentiality protocols were followed.

Questionnaire

An online questionnaire was used with informed consent. The questionnaire was in Arabic, and it contained the definition of mental illness as a disturbance of the mental function that was diagnosed by a healthcare professional. Also, it contained questions about the biographical data of the participants such as age, gender, and socioeconomic status. The second section of the questionnaire was a scale called community attitudes toward mental illness (CAMI), which was validated in Arabic in another study [24]. The Cronbach's alpha of the scale was 0.776.

The CAMI scale was divided into four parts (authoritarianism, benevolence, social restrictiveness, and community mental health ideology), and each part contained 10 questions. Authoritarianism was about questions related to whether or not the mentally ill are inferior and in need of supervision. Benevolence contained questions related to the attitude of kindness towards the mentally ill. The social restrictiveness assessed whether the mentally ill were a source of danger to society or not, and the community mental health scale was related to the inclusion of the mentally ill in the community.

Each item in the scale had five possible answers and was assessed in a five-point system. The answers were as follows: strongly agree (5 points), agree (4 points), neither agree nor disagree (3 points), disagree (2 points), and strongly disagree (1 point); with a higher score meaning more stigmatization. The questions that are stated negatively were pointed in a reversed manner.

Inclusion and exclusion criteria

The inclusion criteria of the study included people aged 18-65 years old whose place of residency during the time of the study was Al-Ahsa; while anyone who was younger than 18 years of age, older than 65 years, or not living in Al-Ahsa during the time of the study was excluded.

Ethical approval and statistical analysis

King Fahad Hospital Hofuf (IRB KFHH No.: H-05-HS-065), the facility responsible for ethical approvals in the region, issued ethical approval (KFHH RCA NO: 07-EP-2021). The data were analyzed using SPSS version 26 (IBM SPSS Statistics, Armonk, NY).

## Results

Statistical analysis

The data were analyzed using SPSS Statistics for Windows, version 26 (IBM SPSS Statistics, Armonk, NY). The 95% confidence interval was used, and a P-value of less than 0.05 was used to determine significance. An independent sample t-test was used to determine the difference between two means and one-way ANOVA was used to compare the means of more than two groups. Multiple linear regression was used with the stigma score as the dependent variable and the biographical data as independent variables.

Sample

The sample size was 888. One participant was excluded due to refusal to give consent for participation in the study, seven were excluded due to being outside of the determined age range, and 122 were excluded because their place of residency was outside of the Al-Ahsa region, leaving a total of 758 participants. Participants aged between 30 and 49 years of age formed most of the sample with a total of 44%, those aged 18-29 years old formed 36.3%, and male participants were 66.4% of the sample. The majority of the participants had a university level of education (77.2%), and 67.4% of them were married. Of the participants, 7.7% were diagnosed with mental illness, 28.8% had a family member diagnosed with a mental illness, and 45% knew someone outside of their family that was diagnosed with a mental illness. The rest of the biographical data are in Table 1.

**Table 1:** Biographical data of participants.

Category	Item	Frequency	Percent
Educational level	Middle school	15	2%
	Primary	1	0.10%
	Secondary	121	16%
	Technical	36	4.70%
	University	585	77.20%
Career	Employee	309	40.80%
	Freelancer	29	3.80%
	Healthcare employee	70	9.20%
	Retired	84	11.10%
	Student	198	26.10%
	Unable to work	11	1.50%
	Unemployed	57	7.50%
Monthly income	Less than 4,000	262	34.6%
	4,000–7,999	113	14.9%
	8,000–11,999	119	15.7%
	12,000 and above	264	34.8%
Marital status	Married	511	67.4%
	Single	233	30.7%
	Divorced	11	1.5%
	Widower	3	0.4%
Number of spoken languages	One language	324	42.74%
	Two languages	419	55.28%
	More than two languages	15	1.98%
Diagnosed with a mental illness	Yes	58	7.70%
	No	700	92.30%
A family member diagnosed with a mental illness	Yes	218	28.80%
	No	540	71.20%
Knows someone outside the family diagnosed with mental illness	Yes	341	45%
	No	417	55%

Stigma score

The scores of all the 758 participants were enrolled. The higher the score, the higher the stigma in the participant. The negatively stated questions were scored in an opposite manner to the rest of the questions. Each question had a score of 1-5. The scores were divided into four categories as presented in Table 2 [11]. A total of 40 questions in the CAMI scale were divided into four subscales (authoritarianism, benevolence, community mental health ideology, and social restrictiveness,) each with 10 questions. The mean of the stigma score was 99.24 ± 15.622, with a minimum of 53 and a maximum of 176. The means of the subscales were as follows: authoritarianism (26.18 ± 4.420), benevolence (23.26 ± 4.762), community mental health ideology (24.66 ± 5.896), and social restrictiveness (25.13 ± 4.6).

**Table 2:** Categories of stigma score levels.

Score	Category	Frequency
≤2	Low stigma Level	97 (12.8%)
>2-3	Medium-low stigma level	600 (79.16%)
>3-3.9	Medium-high stigma level	60 (7.92%)
>3.9	High stigma level	1 (0.13%)

T-test

T-test of independence was used to compare the means of stigma score between two groups. There was no significant difference between males (mean = 99.03 ± 16.016) and females (mean = 99.66 ± 14.836) in the mean of stigma score (t(756) = −0.53, P = 0.596). However, there was a significant difference between those who were diagnosed with mental illness (mean = 93.05 ± 19.935) and those who were not (mean = 99.75 ± 15.115) (t(756) = −3158, P = 0.002), indicating that people who were not diagnosed with mental illness had higher stigma. Similarly, there was a significant difference between those who had a family member diagnosed with a mental illness (mean = 96.56 ± 15.658) and those who did not (mean = 100.32 ± 15.492) (t(756) = −3012, P = 0.003), and those who knew someone outside the family diagnosed with mental illness (mean = 97.46 ± 15.871) and those who did not (mean = 100.69 ± 15.282) (t(756) = −2.845, P = 0.005).

One-way ANOVA

One-way ANOVA with post hoc Scheffe (using a P-value of less than 0.05 as significant) was used to compare the means of stigma score between more than two groups. Between the age groups, there was a statistically significant difference in the mean of the stigma score (F(2,755) = 4.019, P = 0.018), showing that people aged 50-65 years had significantly higher stigma than those aged 18-29 years. However, when comparing participants aged 30-49 to people aged 18-29 and people aged 50-65, there was no statistically significant difference between people aged 30-49 and the other two age groups in the mean of the stigma score. There was also a significant difference among people with different levels of education (F(4,753) = 2.680, P = 0.031); however, since there was only one participant with a primary level of education, it was excluded to perform a post hoc test, with the new results (F(3,753) = 3.383, P = 0.018) showing that people with a university level of education had significantly less stigma than those with a secondary level of education. For the career groups (F(6,751) = 5.296, P = 0.0001), healthcare workers had significantly less stigma than the other groups, and there was no significant difference between the non-healthcare careers. Moreover, people with an income of 12,000 or more had significantly less stigma than those with an income of 8,000-11,999 (F(3,754) = 4.157, P = 0.006). There was no significant difference in the mean of the stigma score between marital status groups (F(3,754) = 1.061, P = 0.365). For spoken languages, there was a statistically significant difference between the spoken languages’ groups in the mean of stigma score (F(2,755) = 11.911, P = 0.0001); post hoc Scheffe showed that the difference is only significant between people who spoke only one language and those who spoke two languages, where the first group had a higher mean of the stigma score.

Regression

Multiple linear regression was used with the stigma score as a dependent variable and biographical data as the independent variable. The biographical data used were age (β = 3.451), monthly income (β = −2.218), spoken languages (β = −4.022), being diagnosed with a mental illness (β = 5.096), and having a family member diagnosed with a mental illness (β = 2.985). To account for a confounding variable for certain factors, multiple linear regression was used with two independent factors at a time to measure the adjusted β for a certain variable by another one. It was found that when adjusted by career, the β of monthly income increased by 37.25% (β = −1.071, adjusted β = −0.672) and the significance level has changed (P = 0.016, adjusted P = 0.161), which indicates that career is a confounder for monthly income (see Appendix).

## Discussion

This study aims to assess the level of mental stigma among the population of the Al-Ahsa region in Saudi Arabia, which can help in determining the need to find ways to reduce this stigma. The study results indicate that in contrast to the hypothesis, the majority of the population had a level of stigma that is low or medium-low (91.96%). These results are reflected on each subscale individually, which indicates that in each aspect of stigma measured, the stigma level was almost equivalent. When considering the background knowledge in the literature regarding the attitude towards mental illness in Arabic countries, including Saudi Arabia, we hypothesized that the same would apply to our participants, and stigma would be high [5,8,14,24]. A study from Lebanon found that only 32.2% of the participants had a high positive behavior score towards the mentally ill [24]. On the other side, a similar study from New Zealand using the CAMI scale in addition to other scales found similar results to this research where most participants showed a positive or a very positive attitude towards people diagnosed with mental illness [11]. The factors that were found to predict stigma level in each of these studies were similar, and also in line with the predicting factors in this study. For Lebanon, the factors affecting stigma were state of residency, age, knowing someone diagnosed with mental illness, and gender. In New Zealand, the factors were age, ethnicity, socioeconomic status, and knowing someone diagnosed with mental illness.

The factors in this study that had an impact on the stigma level were age, level of education, career, income, number of spoken languages, being diagnosed with a mental illness, and knowing someone who was diagnosed with a mental illness. On the other hand, gender and marital status did not bear a significant impact on the score. In other studies, knowledge of the mental illness, higher education, and having an experience with a person diagnosed with mental illness were predictors for mental stigma [14,24]. However, for gender, there was a variety of findings in the literature regarding its significance as a predictor of stigma [8,10,11].

Regarding age groups, the data suggested that there is a positive correlation between age and stigma scores. People aged 18-29 years had the lowest stigma scores, with a significant increase only observed when comparing them to those aged 50-65 years. However, when comparing those in the age group of 30-49 years to the other groups, it is noticed that their scores were in the middle, but not significantly lower or higher than the other two groups. This indicates that the increase in stigma takes a steady rise rather than an acute one. This is very similar to the other studies conducted in the United Kingdom, New Zealand, and Canada, where there was a significant difference between those aged 65 years and above and the rest of the age groups, and no significant difference among the age groups younger than 65 years old [10,11,13]. Additionally, a study from Germany that addressed the change of perception towards mental stigma found that it has improved over the years, especially in the younger age group [9]. These findings indicate that stigma could improve with time, as younger generations adopt better attitudes towards the mentally ill.

It was also found that there was a decrease in the level of stigma with the increase of the level of education, as participants with a university level of education had lower stigma scores in comparison to those with lower levels of education. However, in previous studies, knowledge and stigma were not always related to each other [10]. One additional finding was that the number of spoken languages by the participant did have a significant role on the stigma score, as people who spoke more languages had better results. This factor, however, was not found to be associated with the level of education of the participants, indicating that having a higher level of education was not the reason behind this finding. It could be that speaking more languages, while not meaning having higher education, may mean that the person is more exposed to other cultures, such as that of English-speaking countries, and open to their views. Such exposure could explain how speaking foreign languages can reduce the stigma level. To our knowledge, language was not evaluated as a predictor for stigma in other studies besides this one, and this topic needs to be further evaluated.

Furthermore, being a healthcare worker was found to be associated with a lower stigma level when compared to other industries. Healthcare workers are more educated about mental illness and more exposed to those suffering from it than the general public, which could be an explanatory factor to this finding. However, this topic needs further investigation.

In addition, having a higher income resulted in a lower stigma score. Nevertheless, in this study, 17% of those earning a high income are healthcare workers. Also, it was found that a career is a confounder for monthly income, which might indicate that those with higher income had better results because of the portion of them that were healthcare workers.

Moreover, those who were diagnosed with mental illness or knew someone who was diagnosed with mental illness have shown lower levels of stigma. Supposedly, being diagnosed with mental illness or knowing someone diagnosed with it could lead to a better understanding of mental illness and showing more sympathy towards those who suffered from it. This is a recurring theme in the literature, where having an experience with mental illness is an important factor that can positively affect stigma [11,24].

The findings of this study must be put into consideration with the limitations that have been encountered. The sample size was limited by the demographic area, and it was difficult to reach regions outside of Al-Ahsa and represent the Saudi population. Adding to the previous point, it was only possible to include the CAMI scale and only measure the stigma level, which was directly related to the aim of this study. Other scales that measure the knowledge of mental illness or the behaviors toward the mentally ill were not included; however, many studies in the literature discussed these points in Saudi Arabia, which limited their necessity in this study. Another important point to mention was that this study aimed to measure the stigma level towards mental illness in general and did not specify specific mental disorders. It was found in other studies that the attitude towards mental illness can vary depending on the mental illness [10]. However, this is out of the scope of this study, as the aim was to investigate the participants' points of view and stigma towards mental illnesses in general.

Based on the findings of this study, further studies need to consider the possible variables and predictors for mental stigma. This can aid in making campaigns that target these variables for a better outcome. For example, as it has been found in this study and many others, the youth is a better target than other age groups for mental stigma awareness campaigns [6,9-11,13]. Also, it is recommended that these campaigns focus on reducing mental stigma rather than aiming to increase mental health awareness in general.

## Conclusions

This study measures the level of mental stigma among the population of the Al-Ahsa region in Saudi Arabia. The main finding was that most of the population (91.96%) had a low to medium-low level of stigma. The main variables found to affect the level of stigma in this study were age, career, number of spoken languages, being diagnosed with a mental illness, and knowing someone who was diagnosed with a mental illness. An increase in age was associated with a higher stigma. Healthcare workers had significantly less stigma than the other industries. Speaking more languages, being diagnosed with a mental illness, and knowing someone who was diagnosed with a mental illness were associated with less stigma level. It is recommended that future campaigns address these factors and aim specifically to improve mental stigma rather than increase mental knowledge in general. Mental stigma has a great impact on the person diagnosed with mental illness, as it can prevent the patient from seeking help, delay the treatment and recovery, lead to isolation, unemployment, and suicidal ideation.

## Appendices

**Table 3:** Multiple linear regression. R square = 7.5%; constant = 90.234; P-value <0.05 was considered significant. CAMI, community attitudes toward mental illness.

Model: Linear regression with stigma score (CAMI) as the dependent variable		Unstandardized β	Standardized β	P-value
	Constant	90.234		.000
	Age	3.506	.164	.001
	Career	−.081	−.007	.860
	Monthly income	−2.272	−.186	.000
	Have you been diagnosed with a mental illness before (by a specialized healthcare professional)	5.113	.087	.015
	Have any of your family members been diagnosed with a mental illness before (by a specialized healthcare professional)	2.985	.087	.015
	What are the languages you speak	−4.044	−.137	.000

**Table 4:** Multiple linear regression for accounting for confounding variables.

Linear regression and adjusted β for monthly income, spoken languages, career, and age			
	Unstandardized β	Adjusted unstandardized β	Difference
Monthly income	−1.071	By career (−0.672)	37.25%
		By education (−1.0)	6.63%
Languages spoken	−5.148	By education (−5.001)	2.86%
		By career (−4.757)	7.6%
		By income (−5.369)	4.29%
Career	1.182	By education (1.145)	3.13%
